# Supplementary material for: Current and Future Spatial Distribution of the Aedes aegypti in Peru Based on Topoclimatic Analysis and Climate Change Scenarios
Source: Insects. 2025 May 2;16(5):487. doi: 10.3390/insects16050487 (PMC12112751; doi:10.3390/insects16050487)
Supplement: Supplementary file 1 [file insects-16-00487-s001.zip › insects-3562624-supplementary.pdf]

Figure S1. Variable selection with Pearson correlation analysis

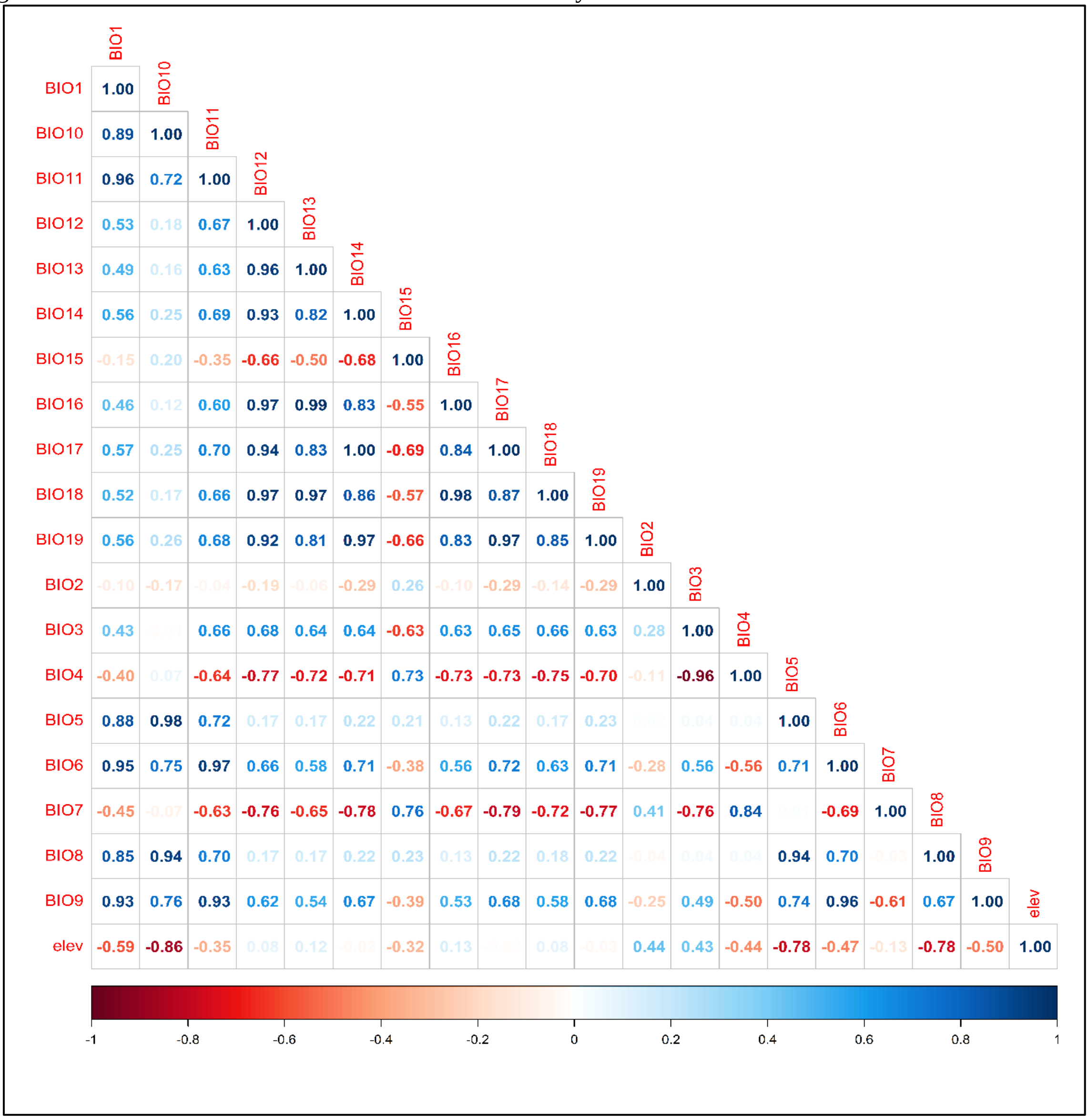

Table S1. Correlation of variables selected for modeling with Maxent (Correlation <0.8)

Means, standard deviations, and correlations with confidence intervals

Variable	*M*	*SD*	1	2	3	4	5	6	7	8	9	10	11	12
1. BIO1	23.72	2.28												
2. BIO10	24.91	2.09	.89**<br>[.88, .89]											
3. BIO11	22.51	2.73	.96**<br>[.96, .96]	.72**<br>[.71, .73]										
4. BIO13	173.11	114.50	.49**<br>[.47, .52]	.16**<br>[.13, .19]	.63**<br>[.61, .64]									
5. BIO14	56.44	52.91	.56**<br>[.54, .58]	.25**<br>[.23, .28]	.69**<br>[.67, .70]	.82**<br>[.81, .83]								
6. BIO15	61.23	49.51	-.15**<br>[-.18, -.12]	.20**<br>[.17, .22]	-.35**<br>[-.37, -.32]	-.50**<br>[-.52, -.48]	-.68**<br>[-.69, -.66]							
7. BIO2	10.90	1.00	-.10**<br>[-.12, -.07]	-.17**<br>[-.19, -.14]	-.04**<br>[-.07, -.01]	-.06**<br>[-.09, -.04]	-.29**<br>[-.31, -.26]	.26**<br>[.23, .28]						
8. BIO3	79.27	9.52	.43**<br>[.41, .45]	-.01<br>[-.04, .01]	.66**<br>[.64, .68]	.64**<br>[.62, .65]	.64**<br>[.62, .65]	-.63**<br>[-.64, -.61]	.28**<br>[.25, .30]					
9. BIO4	100.41	78.91	-.40**<br>[-.42, -.38]	.07**<br>[.04, .10]	-.64**<br>[-.66, -.63]	-.72**<br>[-.73, -.71]	-.71**<br>[-.73, -.70]	.73**<br>[.72, .74]	-.11**<br>[-.14, -.08]	-.96**<br>[-.97, -.96]				
10. BIO5	30.60	1.98	.88**<br>[.87, .89]	.98**<br>[.98, .98]	.72**<br>[.71, .74]	.17**<br>[.14, .20]	.22**<br>[.19, .25]	.21**<br>[.18, .23]	.02<br>[-.01, .04]	.04*<br>[.01, .06]	.04**<br>[.01, .07]			
11. BIO7	13.93	1.92	-.45**<br>[-.47, -.43]	-.07**<br>[-.10, -.04]	-.63**<br>[-.65, -.62]	-.65**<br>[-.67, -.63]	-.78**<br>[-.79, -.77]	.76**<br>[.75, .77]	.41**<br>[.39, .44]	-.76**<br>[-.77, -.74]	.84**<br>[.83, .85]	.01<br>[-.02, .04]		
12. BIO8	24.57	2.31	.85**<br>[.84, .86]	.94**<br>[.94, .95]	.70**<br>[.69, .71]	.17**<br>[.14, .19]	.22**<br>[.19, .25]	.23**<br>[.21, .26]	-.04**<br>[-.07, -.01]	.04**<br>[.01, .07]	.04**<br>[.01, .07]	.94**<br>[.94, .95]	-.03<br>[-.06, .00]	
13. elev	483.80	464.49	-.59**<br>[-.60, -.57]	-.86**<br>[-.87, -.86]	-.35**<br>[-.38, -.33]	.12**<br>[.09, .14]	-.02<br>[-.05, .00]	-.32**<br>[-.35, -.30]	.44**<br>[.42, .47]	.43**<br>[.41, .45]	-.44**<br>[-.46, -.42]	-.78**<br>[-.80, -.77]	-.13**<br>[-.16, -.11]	-.78**<br>[-.79, -.76]

Note. M and *SD* are used to represent mean and standard deviation, respectively. Values in square brackets indicate the 95% confidence interval for each correlation. The confidence interval is a plausible range of opulation correlations that could have caused the sample correlation (Cumming, 2014). * indicates $p < .05$. ** indicates $p < .01$.

Table S2. Areas of *A. aegypti* distribution categorized by department within Peru

Departments	Total Area	Total population	High suitable area for A. aegypti		Human population affected	
	Km^2	Thousands	Km^2	%	Thousands	%
Amazonas	39236.61	426806	7,532.75	19.2	81939	19.20
Ancash	35875.16	1180638	1,917.65	5.35	63109	5.35
Apurímac	21114.15	430736	158.09	0.75	3225	0.75
Arequipa	63131.98	1497438	2,628.39	4.16	62343	4.16
Ayacucho	43508.57	668213	1,637.81	3.76	25154	3.76
Cajamarca	32934.01	1453711	7,922.91	24.1	349718	24.06
Callao	124.098	1129854	84.79	68.3	771973	68.33
Cusco	72073.97	1357075	7,065.75	9.8	133040	9.80
Huancavelica	22061.97	365317	32.39	0.15	536	0.15
Huánuco	37516.4	760267	2,893.98	7.71	58646	7.71
Ica	20901.12	975182	4,162.33	19.9	194202	19.91
Junín	44123.59	1361467	5,831.74	13.2	179943	13.22
La Libertad	25246.04	2016771	3,961.90	15.7	316495	15.69
Lambayeque	14569.03	1310785	10,470.23	71.9	942013	71.87
Lima	34907.29	10628470	1,257.54	3.6	382892	3.60
Loreto	372301.11	1027559	15,598.88	4.19	43053	4.19
Madre de Dios	84175.17	173811	0	0	0	0.00
Moquegua	15769.91	192740	338.92	2.15	4142	2.15
Pasco	23879.45	271904	5,422.28	22.7	61741	22.71
Piura	35336.67	2047954	19,434.85	55	1126356	55.00
Puno	66656.43	1237997	469.2	0.62	8714	0.70
San Martín	51014.11	899648	29,044.47	56.9	512207	56.93
Tacna	15804.76	370974	605.12	3.83	14204	3.83
Tumbes	4475.66	251521	2,456.70	54.9	138060	54.89
Ucayali	104528.6	589110	1,125.29	1.08	6342	1.08

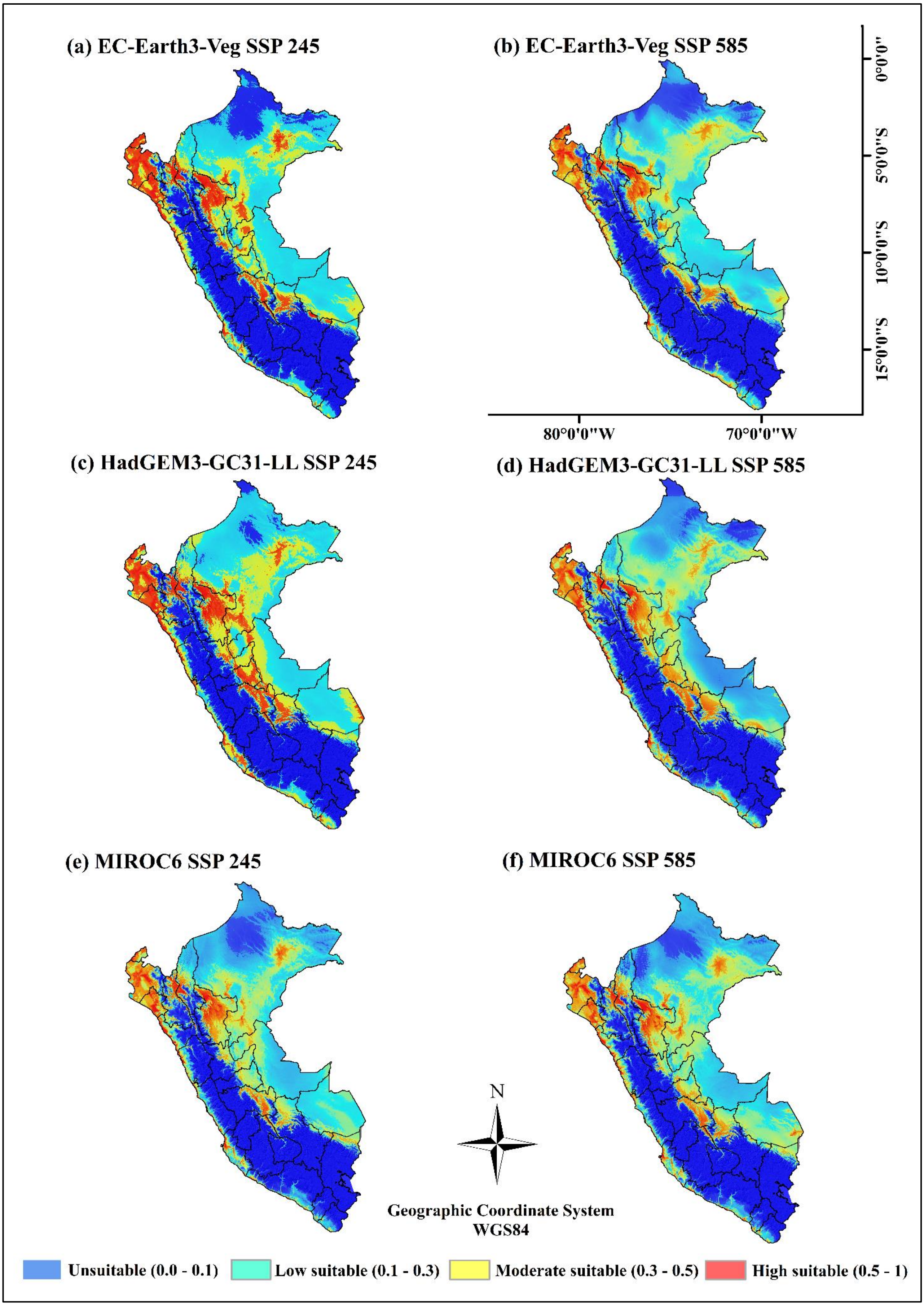

Figure S2. Projected future distribution of *Ae. aegypti* in the year 2070 under both favorable (SSP 245) and unfavorable (SSP 585) climate scenarios

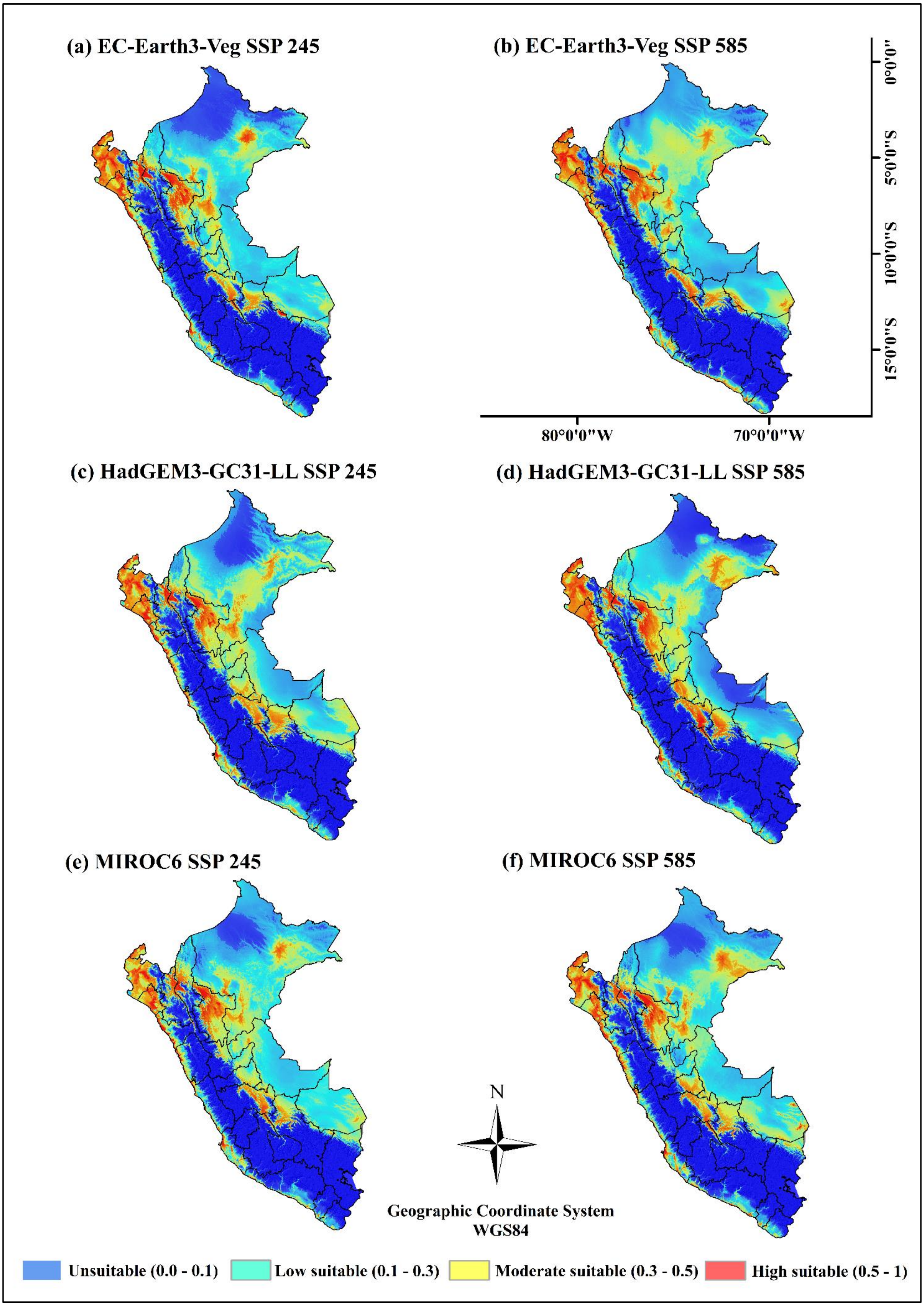

Figure S3. Projected Future distribution of *Ae. aegypti* for the year 2100 under both favorable (SSP 245) and unfavorable (SSP 585).
